# Flexible modelling of spatial variation in agricultural field trials with the R package INLA

**DOI:** 10.1007/s00122-019-03424-y

**Published:** 2019-09-18

**Authors:** Maria Lie Selle, Ingelin Steinsland, John M. Hickey, Gregor Gorjanc

**Affiliations:** 1 Department of Mathematical Sciences, Norwegian University of Science and Technology (NTNU), Trondheim, Norway; 2 The Roslin Institute and Royal (Dick) School of Veterinary Studies, University of Edinburgh, Easter Bush, Edinburgh, UK

## Abstract

***Key message*:**

**Established spatial models improve the analysis of agricultural field trials with or without genomic data and can be fitted with the open-source R package INLA.**

**Abstract:**

The objective of this paper was to fit different established spatial models for analysing agricultural field trials using the open-source R package INLA. Spatial variation is common in field trials, and accounting for it increases the accuracy of estimated genetic effects. However, this is still hindered by the lack of available software implementations. We compare some established spatial models and show possibilities for flexible modelling with respect to field trial design and joint modelling over multiple years and locations. We use a Bayesian framework and for statistical inference the integrated nested Laplace approximations (INLA) implemented in the R package INLA. The spatial models we use are the well-known independent row and column effects, separable first-order autoregressive ($\mathrm{AR1} \otimes \mathrm{AR1}$) models and a Gaussian random field (Matérn) model that is approximated via the stochastic partial differential equation approach. The Matérn model can accommodate flexible field trial designs and yields interpretable parameters. We test the models in a simulation study imitating a wheat breeding programme with different levels of spatial variation, with and without genome-wide markers and with combining data over two locations, modelling spatial and genetic effects jointly. The results show comparable predictive performance for both the $\mathrm{AR1} \otimes \mathrm{AR1}$ and the Matérn models. We also present an example of fitting the models to a real wheat breeding data and simulated tree breeding data with the Nelder wheel design to show the flexibility of the Matérn model and the R package INLA.

**Electronic supplementary material:**

The online version of this article (10.1007/s00122-019-03424-y) contains supplementary material, which is available to authorized users.

## Introduction

In plant breeding, the main goal is to select individuals with the best performance as new market varieties or to select individuals with the best genetic potential as parents of the next generation. To this end, breeders use field trials to estimate genetic and breeding values of individuals. Spatial variation is common in such trials, and if not accounted for it can impact the estimation. There can be several sources of spatial variation in a field trial, such as changes in fertility, watering and soil depth. Other sources of spatial variation that often occur are external influences due to the way plots are treated, for example the effect of drilling, spraying and harvesting. This extraneous variation can be handled by the addition of further effects in a model, such as column or row effects.

Traditionally, spatial variation has been accounted for by using control plots, replications and blocks. These approaches do not account for fine-grained spatial variability, in particular they do not account for dependency between neighbouring blocks and plots within blocks, which can affect the estimation of genetic values. Several models have been proposed to model spatial variation. One of the most widely used is the separable first-order autoregressive ($\mathrm{AR1} \otimes \mathrm{AR1}$) model introduced by Cullis and Gleeson (1991) and extended by Gilmour et al. (1997). It has been shown to fit well in many trials (e.g. Gilmour et al. 1997; Rodríguez-Álvarez et al. 2018). There are other models that can correct for spatial variation. For example, there is a whole class of Gaussian intrinsic models based on the seminal work of Besag and Higdon (1999), which have not gained much traction in plant breeding applications. Much has also been done on smoothing techniques, among which the recent SpATS approach explores two-dimensional smooth surfaces through the use of tensor product P-splines (Rodríguez-Álvarez et al. 2018). Nearest neighbour models are reviewed by Piepho et al. (2008), and the use of spatial kernels is also common (Elias et al. 2018; Mao et al. 2019).

Most of the popular spatial methods in plant breeding use lags between plot locations as a distance, while continuous spatial variation is not commonly addressed. If observations are irregularly spaced, the autoregressive and other models assuming equal spacing are not applicable. However, there are extensions to the autoregressive model, using covariance functions known as the power model and the exponential model (Schabenberger and Gotway 2017). The kernel methods presented in Elias et al. (2018) also use covariance functions based on Euclidean distance between plots.

In this paper, we limit the focus to spatial variation in agricultural field trials such as changes in fertility, watering and soil, not to the spatial variation occurring due to the way plots are treated. We model this spatial variation using different models with publicly available open-source software. We fit the common column and row effects and the separable first-order autoregressive $\mathrm{AR1} \otimes \mathrm{AR1}$ model (Cullis and Gleeson 1991; Gilmour et al. 1997). In addition, we fit a Gaussian random field (Matérn) model to the field trial via the stochastic partial differential equation (SPDE) approach introduced by Lindgren et al. (2011).

For inference, we use the Bayesian numerical approximation procedure known as the integrated nested Laplace approximations (INLA) introduced by Rue et al. (2009) with further developments described in Martins et al. (2013). The method is implemented in the R package INLA where models are fit with the inla() function with the same ease as using the base R functions lm() or glm(). INLA calculates marginal posteriors for all model parameters (fixed and random effects and hyper-parameters) and linear combinations of effects without using sampling-based methods such as Markov chain Monte Carlo (MCMC). It is based on numerical approximations and numerical methods for sparse matrices and is much faster than sampling-based methods (Rue and Martino 2007).

INLA has previously been compared with several other methods for statistical inference. One of these is Mathew et al. (2015) who compared INLA, MCMC (as implemented in the R package MCMCglmm; Hadfield et al. 2010) and restricted maximum likelihood (REML) (as implemented in the ASReml program; Butler et al. 2009), and found that INLA can be used for rapid and accurate estimation of genetic parameters. The computation time for INLA and REML was about the same and significantly shorter than with MCMC, which was also the conclusion of Holand et al. (2013). Huang et al. (2017) compared INLA and REML for spatial models and showed that the performance of INLA–SPDE was comparable to REML. We emphasize that these comparisons are not straightforward because different programs implement different computational methods as well as different models. For example, the R package INLA implements a full Bayesian analysis (using the INLA method), as does the R package MCMCglmm (using the MCMC method), while the ASReml program implements an empirical Bayes analysis (using a two-stage method where first hyper-parameters are estimated and then using these estimates the fixed and random effects are estimated). Gianola et al. (1986) and Sorensen and Gianola (2007) describe these differences in great detail.

The R package INLA is flexible with respect to the field trial design and to including several years and locations in the analysis. For example, it can fit designs beyond the standard lattice design, which we demonstrate with the Nelder wheel design used in forestry (Parrott et al. 2012). For a recent review and comprehensive treatment of the R package INLA, see Bakka et al. (2018) and Krainski et al. (2018).

The objective of this article was to test established spatial models for analysing agricultural field trials using the open-source R package INLA. This R package allows us to fit multi-trial data where designs vary between trials and do not necessarily have to be regular. With a simulation study, we show that the Matérn model performs equally well as the $\mathrm{AR1} \otimes \mathrm{AR1}$ model. Further, using the package enables full Bayesian analysis. We also fitted the models on wheat data from Lado et al. (2013) and on a simulated tree breeding data set with the Nelder wheel design to further demonstrate the flexibility of the Matérn model and SPDE approach implemented in the R package INLA.

## Material and methods

In this section, we present the data for a simulated wheat breeding programme, a real wheat field trial and a simulated tree breeding trial with the Nelder wheel design. We also present the used statistical models, studied cases, how we inferred model parameters and how we evaluated the different models.

### Experimental design and data

#### Simulated wheat data

To evaluate and compare the proposed models, we have simulated a wheat breeding programme and corresponding field trials using the R package AlphaSimR (Faux et al. 2016; Gaynor et al. 2019). The simulation followed closely our previous work (Gaynor et al. 2017; Gorjanc et al. 2018), where we simulated a wheat-like genome and 30 years of a wheat breeding programme with field trials.

The ancestral wheat-like genome had 21 chromosomes, each with 1000 single nucleotide polymorphism markers and 1000 quantitative trait loci. Each year in the breeding programme was based on 100 crosses between 50 parental inbred lines with 100 doubled-haploid lines per cross, resulting in a total 10,000 lines. These were planted in headrows, and the 1000 best individuals were planted in a preliminary yield trial with 0.25 heritability. The 100 best went through a final stage of planting and selection. The 50 best individuals from the preliminary yield trial and the following stages were used as parents in the next year of the breeding programme. Selection was based on phenotype with the exception of the preliminary yield trial in years 20 through 30, where we used the estimated breeding value.

We have focused our attention to the preliminary yield trial, because this stage has low replication, which makes modelling of spatial variation important. The 1000 lines in the preliminary yield trial were planted in two locations, with plots randomly assigned, ensuring that each line was planted once in each location so that the two locations were considered as replicates. The fields in the two locations had the same design, plots arranged in a lattice with 50 rows and 20 columns. The distance between columns was twice as large as the distance between rows causing long and narrow plot shape.

We let the years 1 through 19 serve as burn-in years for the breeding programme, and for years 20 through 30 the plots in the preliminary yield trial were assigned spatially dependent effects. We sampled plot spatial effects from a Matérn model generated via the SPDE approach with a spatial range of 10 units. We varied the proportion of variation due to spatial effects to be $0\%$, $50\%$, or $75\%$ of the residual variance, that is, with $50\%$ a half of variation between plots was due to spatial effects and a half due to other unknown effects (plot residual). More detailed description of the Matérn model and the SPDE approach is given in the “Spatial effect” and “The SPDE approach to spatial modelling” sections. To simulate yield phenotypes, we summed the year, location, individual genetic, spatial dependent plot and independent plot residual effects. We sampled year and location effects from a Gaussian distribution with an expected value of 0 and variance equal to residual variance. Individual genetic effects were based on quantitative trait loci genotypes and corresponding allele substitution effects (Faux et al. 2016; Gaynor et al. 2019). We standardized the yield phenotype before the data analysis, by centring with the mean and scaling with the standard deviation across both locations within the same year.

The reason for simulating spatial effects from the Matérn model generated via the SPDE approach was that this generated realistic geostatistical spatial processes—the true underlying spatial variation in a field is more likely a continuous process rather than discrete process. However, we also simulated spatial effects according to the $\mathrm{AR1} \otimes \mathrm{AR1}$ model. We varied the proportion of variation due to spatial effects to be $0\%$, $50\%$, or $75\%$ of the residual variance, and we set the autocorrelation parameter to be 0.8 in both row and column directions. This autocorrelation corresponds to a range of 10 units.

#### Chilean wheat data

**Fig. 1:**
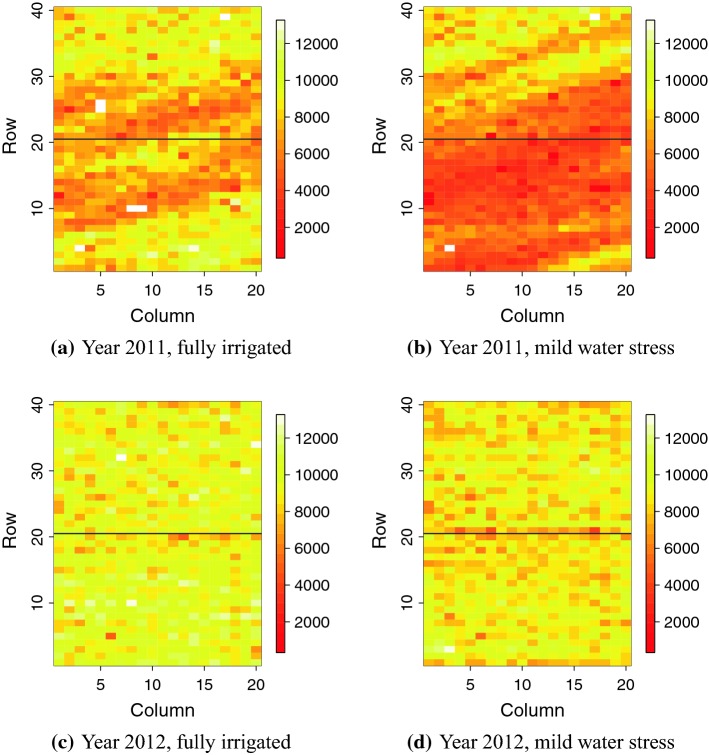
Grain yield in the Chilean wheat data (Lado et al. 2013)

We used parts of the wheat field trial data presented in Lado et al. (2013) and used by Rodríguez-Álvarez et al. (2018) as shown in Fig. 1. The data consisted of 384 advanced lines from wheat breeding programmes in Chile and Uruguay in years 2011 and 2012, and 16 additional lines that were not genotyped. The advanced lines were evaluated in the Santa Rosa region under two different levels of water supply: full irrigation (FI) and mild water stress (MWS). We analysed the total grain yield harvested within each plot.

The experimental design was an alpha-lattice with 20 incomplete blocks, with each block containing 20 genotypes. Two replicates were used for each year and irrigation level, so that each trial had 40 rows and 20 columns, and the lines were assigned the same plot for each year and irrigation level. According to Rodríguez-Álvarez et al. (2018), the replicates were placed such that the first/second 20 rows corresponded to the first/second replicate. This is indicated by the horizontal line in Fig. 1. Plots were twice as long as they were wide and consisted of five rows 2 m long and 0.2 m distance among the rows.

This gave four data sets each with 800 observations. The 384 genotyped lines had 102,324 genome-wide markers. We imputed missing genotypes with the average allele dosage and computed the VanRaden (2008) genomic relationship matrix among the 384 advanced lines. For the 16 lines not genotyped, but with phenotypic observations, we assumed a genomic relationship of zero between themselves and the 384 advanced lines.

One line had missing phenotypic observations for all replicates in 2011, and five other lines had missing phenotypic observation for one replicate each. We standardized the yield phenotype before the data analysis, by centring with the mean and scaling with the standard deviation across all locations for multi-trial models and for each trial separately for the single-trial models.

#### Simulated tree data with the Nelder wheel design

We also simulated data with a design used by tree breeders to test the effect of multiple planting densities on tree growth, known as the Nelder wheel design (Parrott et al. 2012). We chose this particular design to show the flexibility of the R package INLA and the SPDE approach. The Nelder wheel design is circular with rings radiating outward with increasing distance. Spokes connect the centre with the furthest ring, and at the intersections of spokes and rings, a tree is planted. The variable planting densities within a single trial eliminate the need for separate trials for each planting density.

**Fig. 2:**
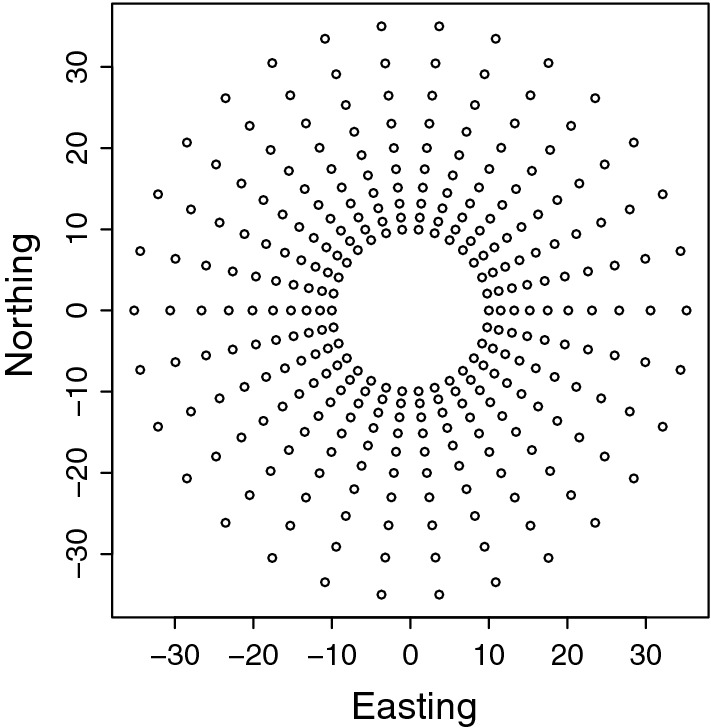
Depiction of the Nelder wheel plot design

In the simulation, we tested 10 different planting densities with 30 planted trees for each density. The inner circle had a radius of 10, and the 9 subsequent circles had a radius of 1.15 times the radius of the previous circle (Fig. 2).

We simulated the phenotype for each tree as a sum of the intercept with a value of 10, the tree density covariate multiplied by a regression coefficient of 10, a spatial effect simulated from a Matérn model using the SPDE approach, and a Gaussian residual with zero mean and variance 0.5. The simulated field (spatial effects) had a variance of 0.5 and a range of 10. There was no other effects to the design, that is, no treatment other than density, since modelling other genetic and environmental effects was illustrated with the simulated wheat data and the Chilean wheat data.

The growing area available to each tree *i* was calculated from:

$$\begin{aligned} \text {Growing area}\,(i) = \frac{\theta r_i^2 (k-k^{-1})}{2}, \end{aligned}$$

where $\theta$ is the angle between rays in radians and $r_i$ is the radius of circle *i*. The factor *k* is 1.15. The planting density was then calculated as the inverse of the growing area.

### Statistical models

We assumed to have *n* plots such that a single field trial was indexed by the rows and columns of an $r \times c$ array. There were $m \le n$ different lines planted in these plots. The observed phenotype $y(s_i)$ was assumed to be a realization of a random variable $Y(s_i)$ in plot coordinates $s_i \in \mathbb {R}^2$, $i = 1,\ldots ,n$. We considered the following general linear model:

1 $$\begin{aligned} y(s_i)|\eta (s_i), \sigma ^2_e \sim \mathscr {N}(\eta (s_i), \sigma ^2_e), \end{aligned}$$

with

2 $$\begin{aligned} \eta (s_i) = \beta _0+ {\varvec{w}}_i{\varvec{\beta }} + g_j + x(s_i), \end{aligned}$$

where $\beta _0$ is an intercept, ${\varvec{\beta }} = (\beta _1,\dots ,\beta _{n_f} )$ is a vector of effects with a known covariate vector ${\varvec{w}}_i$ for plot *i* with $\beta _i \sim \mathscr {N}(0,\sigma ^2_{\beta _i})$ (for example year or location effects), $g_j$ is the genetic effect for individual $j=1,\ldots ,m$ tested in the plot *i* and $x(s_i)$ is the spatial effect for the plot.

#### Genetic effect

We assumed that the genetic effect $g_j$ was a sum of an additive genetic effect (breeding value) $a_j$ and a non-additive (residual) genetic effect $n_j$. For non-additive genetic effects, we assumed an independent prior distribution ${\varvec{n}} \sim \mathscr {N}({\varvec{0}}, {\varvec{I}}_m\sigma ^2_n)$. For additive genetic effects, we assumed that they were fully explained by genome-wide markers such that ${\varvec{a}} \sim \mathscr {N}({\varvec{0}}, {\varvec{A}}\sigma ^2_a)$, where ${\varvec{A}}$ is a relationship matrix. We calculated the relationship matrix as ${\varvec{A}} = {\varvec{Z}}{\varvec{Z}}^T/k$, where ${\varvec{Z}}$ is a column-centred genotype matrix of dimension $m \times p$, *p* is the number of markers, and $k=2\sum _l{q_l(1-q_l)}$ with $q_l$ being allele frequency at marker *l* (VanRaden 2008). An equivalent model for the additive genetic effects was to use the genotype matrix directly, letting ${\varvec{a}} = {\varvec{Z}} {\varvec{u}}$, where ${\varvec{u}}$ are marker effects ${\varvec{u}} \sim \mathscr {N}(0, \mathbf {I}_p \sigma ^2_u)$.

Genome-wide marker data contain substantial amount of shared information among related individuals due to shared genome segments. Therefore, we could compress it to reduce model dimension while retaining information, which saved computation time (e.g. Jolliffe 1986). With singular value decomposition, we obtained:

$$\begin{aligned} {\varvec{Z}} = {\varvec{USV}}^T, \end{aligned}$$

where ${\varvec{U}}$ is a unitary matrix of dimension $(m \times m)$, ${\varvec{S}}$ is the diagonal matrix $(m \times n)$ of singular values and ${\varvec{V}}$ is an $(n \times n)$ matrix of eigenvectors. We used the principal components (the columns of ${\varvec{ZV}}$) corresponding to the largest singular values of ${\varvec{S}}$ and chose $p^*$ components that explained approximately $95\%$ of the variation in ${\varvec{Z}}$. That is, we replaced the ${\varvec{Z}}$ by ${\varvec{Z}}^* = {\varvec{ZV}}(:,1:p^*)$ of dimension $m \times p^*$. The linear predictor from (2) then became:

3 $$\begin{aligned} \eta _{j}(s_i) = \beta _0+ {\varvec{w}}_i{\varvec{\beta }} + {\varvec{z}}^*_{j}{\varvec{u}}^* + n_j + x(s_i), \end{aligned}$$

where ${\varvec{z}}^*_j$ is the *j*th row vector of ${\varvec{Z}}^*$ for individual *j* and ${\varvec{u}}^* \sim \mathscr {N}(0, \mathbf {I}_{p^*} \sigma ^2_{u^*})$ are principal component effects.

#### Spatial effect

We tested the independent row and column effects model, the separable first-order autoregressive ($\mathrm{AR1} \otimes \mathrm{AR1}$) model and a Gaussian random field (Matérn) model via the SPDE approach. The independent row and column model and separable autoregressive model are based on a discretization of the field and model only a finite collection of spatial random variables. For these models, we omit the $s_i$ in $x(s_i)$ and use $x_i$. This is to emphasize that these models use neighbouring plots as opposed to the Gaussian random field which is a continuous spatial process and for which we use the notation $x(s_i)$.

#### Row and column effects model

Row and column effects can model the underlying smooth spatial field as well as external variation due to field management. We assumed:

$$\begin{aligned} x_i = r_i + c_i, \end{aligned}$$

where $r_i \sim \mathscr {N}(0, \sigma ^2_\mathrm{r})$ is the row effect and $c_i \sim \mathscr {N}(0, \sigma ^2_\mathrm{c})$ is the column effect of plot *i*, $i = 1,\ldots ,n$.

#### Separable autoregressive model, $\mathrm{AR1}\otimes \mathrm{AR1}$

The autoregressive model of order 1 (AR1) for the Gaussian vector ${\varvec{x}} = (x_1,\ldots , x_\mathrm{r})$ is defined as:

$$\begin{aligned} x_1&\sim \mathscr {N}(0, \sigma ^2_x /(1- \rho ^2)),\\ x_i | x_{i-1}&\sim \mathscr {N}(\rho x_{i-1}, \sigma ^2_x),\quad i =2,\ldots ,r, \end{aligned}$$

where $$|\rho | < 1$$.

For modelling the influence of neighbouring plots along rows and columns, the autoregressive model in each direction was combined into a two-dimensional first-order separable autoregressive model (Cullis and Gleeson 1991; Gilmour et al. 1997), denoted as $\mathrm{AR1} \otimes \mathrm{AR1}$. In this model the spatial effect vector ${\varvec{x}}$ of length *n* was modelled as:

$$\begin{aligned} {\varvec{x}} \sim \mathscr {N}({\varvec{0}}, {\varvec{\Sigma }}\sigma _x^2), \end{aligned}$$

with ${\varvec{\Sigma }} = {\varvec{\Sigma }}_\mathrm{r} \otimes {\varvec{\Sigma }}_\mathrm{c}$. The matrices ${\varvec{\Sigma }}_\mathrm{r}$ and ${\varvec{\Sigma }}_\mathrm{c}$ are the covariance matrices of first-order autoregressive processes in row and column direction, respectively, and $\otimes$ is the Kronecker product. The model had two dependency parameters, one in each direction, $\rho _\mathrm{r}$ and $\rho _\mathrm{c}$, and a variance parameter $\sigma _x^2$.

#### Gaussian random fields and the Matérn model

In the model described above, the spatial variation was modelled as discrete, meaning that the model only considers data on a fixed field trial layout, possibly allowing the distance between rows to be different from the distance between columns. Assuming a continuous field for the spatial variation is, however, more realistic and allows the spatial variation to be modelled at any observed distance or field trial layout.

Continuously indexed Gaussian random fields play an important role in spatial statistical modelling and geostatistics. In the field $\mathscr {D}\in \mathbb {R}^d$ with coordinates ${\varvec{s}} \in \mathscr {D}$, the continuously indexed Gaussian random field $x({\varvec{s}})$ has a joint Gaussian distribution for all finite collections $\{ x({\varvec{s}}_i)\}$. The Gaussian random field is specified through the mean ${\varvec{\mu }}$ and the covariance matrix ${\varvec{\Sigma }} = C({\varvec{s}}_i,{\varvec{s}}_j)$.

In this study, we used ${\varvec{\mu }}=0$ and the Matérn covariance function, which is the most important covariance function in spatial statistics (Stein 2012). We refer to this Gaussian random field model as the Matérn model. The Matérn covariance function between locations ${\varvec{s}}_i, {\varvec{s}}_j \in \mathbb {R}^d$ was:

4 $$\begin{aligned} C({\varvec{s}}_i, {\varvec{s}}_j) = \frac{\sigma ^2_s}{2^{\nu -1} \Gamma (\nu )}\left( \kappa \Vert {\varvec{s}}_j - {\varvec{s}}_i \Vert \right) ^{\nu } K_{\nu }\left( \kappa \Vert {\varvec{s}}_j - {\varvec{s}}_i \Vert \right) , \end{aligned}$$

where $K_{\nu }$ is the modified Bessel function of the second kind and order $\nu >0$. The parameter $\kappa$ can be expressed as $\kappa = \sqrt{8\nu }/\rho$, where $\rho >0$ is the range parameter describing the distance where the correlation between two points was near 0.1, and $\sigma ^2_s$ is the marginal variance. The parameter $\nu$ determined the mean-square differentiability of the field. The SPDE approach is a computationally efficient way to fit the Gaussian random field (Matérn) model (Lindgren et al. 2011), which we describe in the “The SPDE approach to spatial modelling” section.

#### Prior distributions

We used a full Bayesian approach to estimation which requires prior distributions for all parameters. The model consisted of two layers of parameters. The first layer consisted of fixed and random effects, for which we have specified most prior distributions above. In addition, a Gaussian prior with mean 0 and variance 1000 was assigned to the intercept and covariate effects, meaning $\sigma ^2_{\beta _i} = 1000$. The second layer consisted of the variance/dispersion parameters and other (spatial) parameters controlling the first layer and the likelihood for the data, i.e. all variance parameters, the parameters of the $\mathrm{AR1} \otimes \mathrm{AR1}$ and the Matérn models. For parameters in this layer, which we refer to as the hyper-parameters, we used the default priors of the R package INLA. These are proper, but weak priors. For variance parameters, this was an inverse gamma prior with shape 1 and inverse scale $5 \times 10^{-5}$, which has 95$\%$ percentiles at approximately 0.009 and 0.010. In the separable autoregressive model, the same inverse gamma prior was set for the marginal variance $\sigma ^2_x/(1-\rho ^2)$. The transformed variable $\log ((\rho +1)/(\rho -1))$ was assigned a Gaussian prior with mean 0 and standard deviation 0.15, which has 95$\%$ percentiles at approximately $-\,0.15$ and 0.15 for $\rho$. Priors for the Matérn model were specified for the parameters $\kappa$ and $\tau$ that control spatial range and variance; see the “The SPDE approach to spatial modelling” section. We used the default joint Gaussian prior on $\log (\kappa )$ and $\log (\tau )$ with mean 0 and identity covariance matrix, so that $\log (\kappa )$ and $\log (\tau )$ were independent (Blangiardo and Cameletti 2015) and automatically scale to the size of the field.

### Case studies

#### Simulation study

We fitted the model (1) with two versions of the linear predictor (3) to the preliminary yield trial of each simulated breeding programme—without and with genome-wide markers. The two linear predictors were:

5 $$\begin{aligned}&\eta (s_{i}^k) = \beta _0+ {\varvec{w}}_i{\varvec{\beta }} + x(s_{i}^k) + n_j, \end{aligned}$$

6 $$\begin{aligned}&\eta (s_{i}^k) = \beta _0+ {\varvec{w}}_i{\varvec{\beta }} + x(s_{i}^k) + {\varvec{z}}^*_{j}{\varvec{u}}^*, \end{aligned}$$

where $\beta _0$, ${\varvec{w}}_i{\varvec{\beta }}$, $g_j$, ${\varvec{z}}^*_{j}{\varvec{u}}^*$ were as described as in the “Statistical models” section. The linear predictors differed in that model (5) assuming that individuals were genetically independent, whereas model (6) used genome-wide marker data to model the genetic dependency. The linear predictors included both trials simultaneously. The *k* in $s_{i}^k$ indicated that the plot coordinates $s_i$ were in field *k*, where $k=1,2$, and a fixed effect of location was included in ${\varvec{w}}_i{\varvec{\beta }}$. Otherwise, the two locations were assumed to be independent realizations from the same distribution, and we used all three spatial models described in the “Spatial effect” section to fit spatial variation. We also fitted a model where the spatial effect was omitted, which we denoted as the NoSpatial model. Since the distance between columns was twice as large as the distance between rows, we accounted for this with the Matérn model, by appropriately scaling the column coordinates. The matrix $Z^*$ was constructed using $p^*=500$ principal components of the singular value decomposition of the centred genotype matrix *Z*.

#### Chilean wheat data

Using the data sets from the four trials (Lado et al. 2013) presented in the “Experimental design and data” section, we fitted the model (1) with different versions of the linear predictor (3). The four linear predictors were:

$$\begin{aligned} \begin{array}{lll} \eta (s_i) = \beta _0 + x(s_i) &{}\quad + n_j, &{}\quad \text {W1: wheat model 1} \\ \eta (s_{i}) = \beta _0 + x(s_{i}) &{}\quad + {\varvec{z}}^*_{j}{\varvec{u}}^* + n_j, &{}\quad \text {W2: wheat model 2} \\ \eta (s_{i}^k) = \beta _k + x(s_{i}^k) &{}\quad + n_j, &{}\quad \text {W1M: use all trials} \\ \eta (s_{i}^k) = \beta _k + x(s_{i}^k) &{}\quad + {\varvec{z}}^*_{j}{\varvec{u}}^* + n_j, &{}\quad \text {W2M: use all trials} \end{array} \end{aligned}$$

where $\beta _0$, $x(s_i)$, ${\varvec{z}}^*_{j}{\varvec{u}}^*$, and $n_j$ are as described in the “Statistical models” section. As with the simulation study, we used the three spatial models described above and the NoSpatial model. The linear predictors W1M and W2M included all four trials simultaneously, and therefore the intercept $\beta _k$, $k = 1,\ldots ,4$, was trial specific to capture fixed year and irrigation effects. Further, the *k* in $s_{i}^k$ indicated that the plot coordinates $s_i$ were in field *k*. The four trials in Fig. 1 showed quite different spatial patterns with respect to dependency in distance and variance, so it was not reasonable to assume that they were realizations from the same distribution. However, assigning separate variance and parameters controlling the spatial dependency to each trial increased the number of hyper-parameters considerably. We therefore modelled the spatial effect in the trials from 2011 as independent realizations from the same underlying distribution, and the same for the 2012 trials, because these showed most similar behaviour. This gave two sets of spatial parameters in the model, one set for the 2011 trials and one set for the 2012 trials. We emphasize that this decision was driven by observation of the data. The matrix $Z^*$ was constructed using $p^*=280$ principal components of the singular value decomposition of the centred and scaled genotype matrix *Z*.

#### Nelder wheel plot

To analyse the simulated tree data, we fitted the model (1) with the following linear predictor:

$$\begin{aligned} \eta (s_{i}) = \beta _0 + w_i \beta + x(s_{i}), \end{aligned}$$

where $\beta _0$ is the intercept, $\beta$ is a density effect, and a Matérn model is assumed for the spatial effect $x(s_{i})$. We also fitted a model where the spatial effect was omitted.

### SPDE, inference and evaluation of case studies

#### The SPDE approach to spatial modelling

Modelling with Gaussian random fields is computationally challenging because they give rise to dense precision matrices that are numerically expensive to factorize in the estimation procedures (Rue and Held 2005). Gaussian Markov random fields do not incur this penalty because they have a sparse precision matrix due to their Markov property. Lindgren et al. (2011) showed how to construct an explicit link between (some) Gaussian random fields and Gaussian Markov random fields by showing that the approximate weak solution of the SPDE:

7 $$\begin{aligned} \begin{aligned}&(\kappa ^2 - \Delta )^{\alpha /2} x({\varvec{s}}) = \mathscr {W}({\varvec{s}}), \\&{\varvec{s}} \in \mathbb {R}^d, \alpha = \nu + d/2,\quad \kappa> 0,\;\; \nu >0, \end{aligned} \end{aligned}$$

is a Gaussian random field with Matérn covariance function as given in (4). Here, $\mathscr {W}(\cdot )$ is the Gaussian white noise, $\Delta$ is the Laplacian, $\alpha$ is a smoothness parameter, $\kappa$ is the scale parameter in (4), *d* is the dimension of the spatial domain and $\tau$ is a parameter controlling the variance. The parameters of Matérn covariance are linked to the SPDE through:

$$\begin{aligned} \sigma _s^2 = \frac{\Gamma (\nu )}{\Gamma (\alpha ) (4\pi )^{d/2}\kappa ^{2\nu } \tau ^2} \end{aligned}$$

where $\nu = \alpha - d/2$, and we use $\alpha =2$ and $d = 2$.

A Gaussian Markov random field approximation described in Lindgren et al. (2011) is enabled by solving the SPDE in (7) by the finite element method. Further details on the SPDE approach to spatial modelling can be found in Lindgren et al. (2011).

#### Bayesian inference with INLA and the R package INLA

Statistical inference is carried out using the INLA method introduced in Rue et al. (2009), which is implemented for use in R (R Core Team 2018) in the R package INLA (available at www.r-inla.org). In this section, we give a short introduction to the class of models known as latent Gaussian models and how INLA can be used to approximate the posterior marginal distributions for such models. For an in-depth description of INLA, useful sources are Rue et al. (2009), Martins et al. (2013) and the recent review by Rue et al. (2017).

The class of latent Gaussian models includes many models, for example generalized linear (mixed) models, generalized additive (mixed) models and spline smoothing methods. Latent Gaussian models are hierarchical models in which observations ${\varvec{y}}$ are assumed to be conditionally independent given a latent Gaussian random field ${\varvec{x}}$ and hyper-parameters ${\varvec{\theta }}_1$, that is, $\pi ({\varvec{y}}|{\varvec{x}}, {\varvec{\theta }}_1) \sim \Pi _{i \in \mathscr {I}} \pi (y_i|x_i, {\varvec{\theta }}_1)$. The latent field ${\varvec{x}}$ includes both fixed and random effects and is assumed to be Gaussian-distributed given hyper-parameters ${\varvec{\theta }}_2$, that is, $\pi ({\varvec{x}}|{\varvec{\theta }}_2) \sim \mathscr {N}({\varvec{\mu }}({\varvec{\theta }}_2), {\varvec{\Sigma }} ({\varvec{\theta }}_2))$. The parameters ${\varvec{\theta }} = ({\varvec{\theta }}_1, {\varvec{\theta }}_2)$ are known as hyper-parameters and control the Gaussian field and the likelihood for the data. These are usually variance (dispersion) parameters for simple models, but can also include other parameters, for example autocorrelation. The hyper-parameters must also be assigned a prior density to completely specify the model.

The main aim of Bayesian inference is to estimate the marginal posterior distribution of the variables in the model, that is, $\pi (\theta _j | {\varvec{y}})$ for hyper-parameters and $\pi (x_i|{\varvec{y}})$ for location parameters. INLA computes approximations to these densities quickly and with high accuracy. Laplace approximations are applied to integrals that are Gaussian or close to Gaussian, and for non-Gaussian problems, conditioning is done to break down the approximations into smaller sub-problems that are almost Gaussian.

For the computations in INLA to be both quick and accurate, the latent Gaussian models have to satisfy some additional assumptions. Since INLA integrates over the hyper-parameter space, the number of non-Gaussian hyper-parameters ${\varvec{\theta }}$ should be low, typically less than 10, and not exceeding 20. Further, the latent field should not only be Gaussian, it must be a Gaussian Markov random field. The conditional independence property of a Gaussian Markov random field yields sparse precision matrices which makes computations in INLA fast due to efficient algorithms for sparse matrices. Lastly, each observation $y_i$ should depend on the latent Gaussian field through only one component $x_i$.

The R package INLA can be installed from within R. It is run using the $\texttt {inla()}$ function with three mandatory arguments: a data frame containing the data, a formula much like the formula for the standard $\texttt {lm()}$ function in R and a string indicating the likelihood family. The default is Gaussian with the identity link. The following call generates an object of type inla:

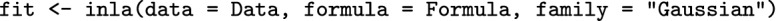



Prior distributions are specified through additional arguments. Several tools to manipulate models and likelihoods exist as described in tutorials at the Web page www.r-inla.org and the books by Blangiardo and Cameletti (2015), Krainski et al. (2018). The $\texttt {R}$ scripts used for the fitted models and the tree breeding simulation are available in Online Resource 1. Specifically we provide R code for all the fitted models to the real wheat data and the simulation and analysis of the tree breeding data with the Nelder wheel design.

Here, we show how to fit an: (1) $\mathrm{Row}+\mathrm{Col}$ model, (2) AR1 row and AR1 col model, (3) $\mathrm{AR1} \otimes \mathrm{AR1}$ model and (4) Matérn model. The data should be stored in a data frame or list. Here, the data frame Data has one row for each observation with columns containing the phenotype, id for each line and row and column in the field. The id for each line is included twice because we want to model the genetic effect with and without genetic markers.

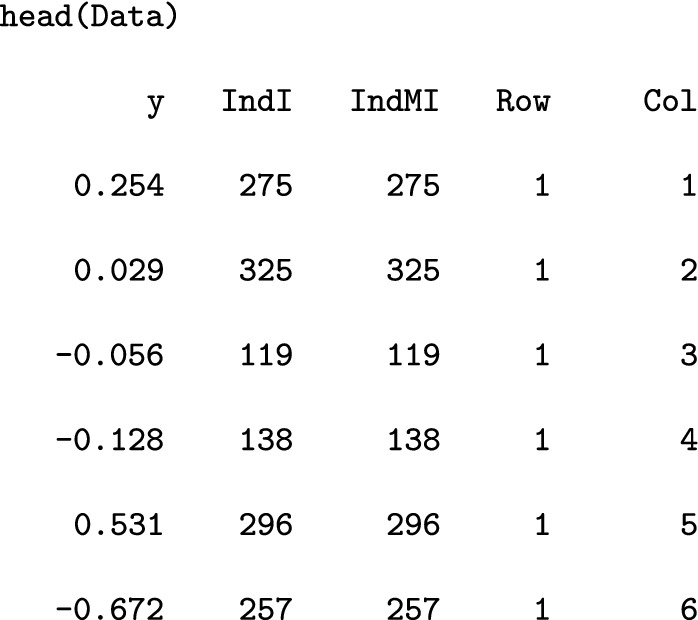



In the formula below, we indicate that each line should be modelled both with an independent normal distributed effect and using marker effects for the markers stored in Gen, the approach described in the “Genetic effect” section.

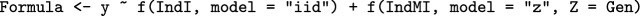



To include a spatial model, one of the following functions can be added to Formula.

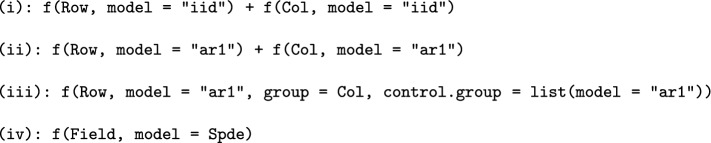



Here, f() indicates a random effect with a specific model. The group argument nests the random effect within each level of the group factor, and the control.group argument specifies the model between the group levels. The models with formula including either of effects (1)–(3) are fitted with the call to inla() as described above. The SPDE approach (4) requires a few additional stages which we show in the full code available in Online Resource 1.

#### Evaluation of model performance

We evaluated the models using the correlation between the true and estimated values, the continuous rank probability score (CRPS), by identifying the top individuals, and the residual variance.

**Fig. 3:**
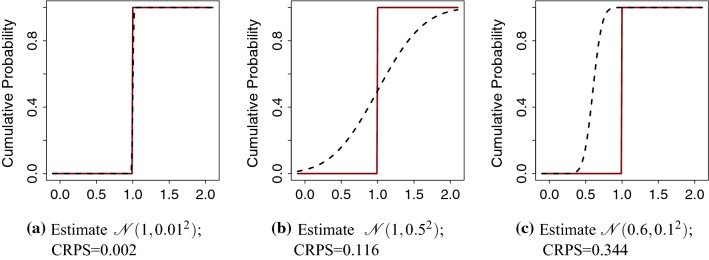
Cumulative distribution function (CDF) of the observation (true value = 1; solid line) and of estimate (dashed line)

We used the CRPS to take into account the whole posterior predictive distribution, that is, to compare the estimated posterior means with the true/observed values while accounting for the uncertainty of estimation. The CRPS is defined as (Gneiting and Raftery 2007):

$$\begin{aligned} \text {CRPS}(F,y) = \int _{- \infty }^{\infty } (F(u) - 1\{y \le u \})^2 \, \mathrm{d}u, \end{aligned}$$

where *F* is the cumulative distribution of the estimator of interest and *y* is the observed value. The CRPS is negative oriented, so the smaller the CRPS the closer the estimated value is to the observed/true value. For readers not familiar with the CRPS, three plots in Fig. 3 show the cumulative distribution functions for estimates and the observed value of 1.0. In Fig. 3a, the estimate is close to the true value and the area between the curves is small and so is the CRPS. In Fig. 3b, the estimated mean is equal to the true value, but the large uncertainty due to estimation causes a large area between the curves, and hence a larger CRPS than in Fig. 3a. In Fig. 3c, the uncertainty of the estimation is small, but the estimated mean is further from the true value, causing the area and the CRPS to be large.

For the simulated data, we computed the correlation and the CRPS between true and estimated breeding value. We also quantified how many of the ten best individuals were among the estimated top 100 individuals.

For the real data, we did not know the true breeding value, and it was therefore not possible to validate the estimated breeding values. We therefore focused on the residual variance from each model as a measure of the unexplained variance. This value can be seen as a proxy for the coefficient of determination ($R^2$), a measure on how much of the data variance is explained by a given model (Gelman and Hill 2006).

## Results

In this section, we present the results from the three cases presented in the “Case studies” section. In the results from the simulation study, we compare correlation, CRPS and top ranking of individuals between the spatial models. In the results from the real data, we present estimated genetic variances, marker variances and residual variances and compare these between the different models. In the results from the simulated tree breeding data, we present the posterior distribution of all parameters and the estimated spatial effect.

### Simulation study

This section presents the results from the simulation study. The models were evaluated using the correlation and CRPS between the true and estimated breeding value and using the number of the top ten individuals that were among the top 100 ranked individuals when considering estimated breeding value (posterior mean). In this section, all tables have three scenarios indicating the proportion of environmental variance due to spatially structured variation in the data, 0.00, 0.50 or 0.75, while the total variance was the same. Proportion of spatial variation therefore indicates how much of total environmental variance was due to structured spatial noise and unstructured noise (see the “Simulated wheat data” section).

In this section, we only present the results from data with spatial effects generated from the Matérn model via the SPDE approach. Tables with results for correlation, CRPS and ranking, based on data with spatial effect generated from the $\mathrm{AR1} \otimes \mathrm{AR1}$ model, are given in the Online Resource 2. These results show similar tendencies to the ones presented in this section. Tables showing average estimates for residual variance, genetic and marker variance and other spatial hyper-parameters based on data with spatial effect generated from the Matérn model are given in the Online Resource 2.

In Table 1, the average correlation is presented, in Table 2 the average CRPS is presented, and in Table 3 the average number of the top ten individuals that are among the top 100 ranked individuals is presented. The average was taken over 100 independent realizations of the breeding programme described in the “Simulated wheat data” section. We note that genomic data improved the correlation, CRPS and the average number of the top ten individuals for all models and proportions of spatial variance. We further note that modelling the spatial variation also improved these metrics. Below, we go through each table in detail.

Across all metrics, the Matérn and $\mathrm{AR1} \otimes \mathrm{AR1}$ stand out as best to model the spatial variation. These had the highest correlation when spatial variation was present as seen in Table 1. When there was no spatial variation, the two models did not perform worse than not including a spatial effect. The performance increased as the extent of spatial variation increased. The CRPS results in Table 2 show lower CRPS for the Matérn model and the $\mathrm{AR1} \otimes \mathrm{AR1}$ models compared to the NoSpatial and $\mathrm{Row}+\mathrm{Col}$ models. These results are in line with the correlation results with one exception for the $\mathrm{AR1} \otimes \mathrm{AR1}$ model. We also note an improvement in CRPS with increasing extent of spatial variation.

The average number of the top ten individuals among the top 100 ranked individuals is given in Table 3. The Matérn and $\mathrm{AR1} \otimes \mathrm{AR1}$ models again had better results when there was a spatial variation in the data and when genome-wide markers were used—in this setting there were on average between 6 and 8 of the top ten individuals among the top 100 ranked individuals. As expected, the NoSpatial showed no improvement when the degree of spatial variation was increased and the $\mathrm{Row}+\mathrm{Col}$ model showed only a little improvement with respect to all evaluations.

**Table 1:** Correlation between the simulated true and estimated breeding value in the preliminary yield trial by the proportion of spatial variation, the spatial model and using genome-wide markers

Genome-wide markers	No			Yes		
Prop. of spatial var	0.00	0.50	0.75	0.00	0.50	0.75
NoSpatial	0.39	0.39	0.39	0.62	0.61	0.62
$\mathrm{Row}+\mathrm{Col}$	0.39	0.41	0.42	0.62	0.63	0.64
$\mathrm{AR1}\otimes \mathrm{AR1}$	0.39	0.47	0.56	0.62	0.68	0.74
Matérn	0.39	0.47	0.57	0.62	0.68	0.74

The standard error was around 0.002

**Table 2:** CRPS between the simulated true and estimated breeding value in the preliminary yield trial by the proportion of spatial variation, the spatial model and using genome-wide markers

Genome-wide markers	No			Yes		
Prop. of spatial var	0.00	0.50	0.75	0.00	0.50	0.75
NoSpatial	0.149	0.149	0.149	0.114	0.115	0.114
$\mathrm{Row} + \mathrm{Col}$	0.169	0.142	0.138	0.114	0.113	0.111
$\mathrm{AR1}\otimes \mathrm{AR1}$	0.169	0.127	0.117	0.114	0.108	0.100
Matérn	0.148	0.127	0.117	0.114	0.107	0.099

The standard error was around 0.0002

**Table 3:** Average number of the top ten individuals among the top 100 ranked individuals in the preliminary yield trial by the proportion of spatial variation, the spatial model and using genome-wide markers

Genome-wide markers	No			Yes		
Prop. of spatial var	0.00	0.50	0.75	0.00	0.50	0.75
NoSpatial	3.89	3.82	3.89	6.32	6.41	6.43
$\mathrm{Row}+\mathrm{Col}$	3.89	4.05	4.20	6.33	6.59	6.77
$\mathrm{AR1} \otimes \mathrm{AR1}$	3.89	4.81	5.81	6.32	7.36	8.07
Matérn	3.89	4.80	5.85	6.32	7.38	8.15

The standard error was around 0.05

We also evaluated predictions of breeding values for 1000 doubled-haploid individuals that were genotyped, but not phenotyped. These individuals served to test out-of-sample prediction, which we could perform using estimated genome-wide marker effects. The average correlation between the true and predicted breeding value is presented in Table 4, where $\mathrm{AR1} \otimes \mathrm{AR1}$ and Matérn again had the highest correlation. For the CRPS in Table 5, we see a similar trend as for the phenotyped individuals; however, the improvement with the higher degree of spatial variation is now less dominant. Finally, the average number of the top ten individuals among the 100 ranked individuals is given in Table 6. These results improved with the Matérn and $\mathrm{AR1} \otimes \mathrm{AR1}$ model and with the increasing spatial variation. The results for the non-phenotyped doubled-haploid lines showed lower correlation, higher CRPS and lower number of the top ten individuals captured than in the preliminary yield trial. This is expected as we had not observed any phenotype data on the doubled-haploid lines.

**Table 4:** Correlation between the simulated true and predicted breeding value for the non-phenotyped doubled-haploid lines by the proportion of spatial variation and the spatial model

Prop. of spatial var	0.00	0.50	0.75
NoSpatial	0.36	0.36	0.36
$\mathrm{Row}+\mathrm{Col}$	0.36	0.37	0.38
$\mathrm{AR1}\otimes \mathrm{AR1}$	0.36	0.42	0.47
Matérn	0.36	0.42	0.48

The standard error was around 0.004

**Table 5:** CRPS between the simulated true and predicted breeding value for the non-phenotyped doubled-haploid lines by the proportion of spatial variation and the spatial model

Prop. of spatial var	0.00	0.50	0.75
NoSpatial	0.128	0.128	0.129
$\mathrm{Row}+\mathrm{Col}$	0.128	0.128	0.127
$\mathrm{AR1} \otimes \mathrm{AR1}$	0.128	0.126	0.122
Matérn	0.128	0.126	0.122

The standard error was around 0.00004

**Table 6:** Average number of the top ten individuals among the top 100 ranked individuals for the non-phenotyped doubled-haploid lines by the proportion of spatial variation and the spatial model

Prop. of spatial var	0.00	0.50	0.75
NoSpatial	3.37	3.38	3.35
$\mathrm{Row}+\mathrm{Col}$	3.37	3.51	3.60
$\mathrm{AR1} \otimes \mathrm{AR1}$	3.37	3.99	4.67
Matérn	3.37	3.97	4.75

The standard error was around 0.06

### Chilean wheat data

**Fig. 4:**
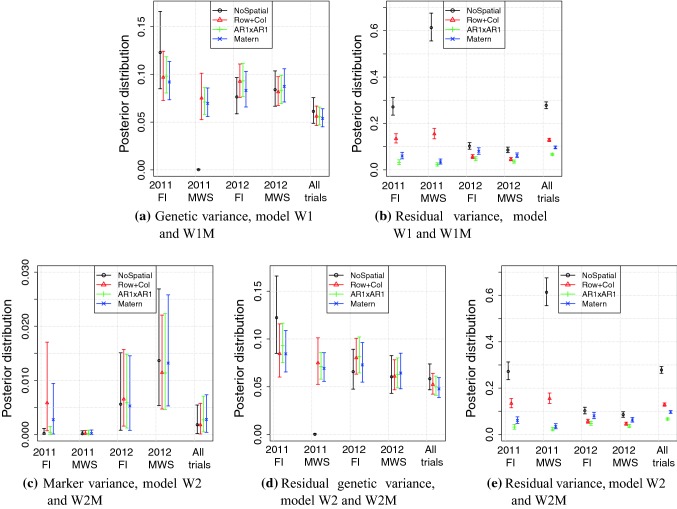
Posterior variances in the models for Chilean wheat data. The top panels are for models that do not use genome-wide marker data (W1 and W1M) and the bottom panels for models that use genome-wide marker data (W2 and W2M)

In this section, we present results from fitting the models W1, W2, W1M and W2M to the Chilean wheat data. We present the estimated genetic variances, marker variances and residual variances from the different spatial models. These are shown in Fig. 4. We also present the posterior predicted phenotype from model W2 for the 2011 trial with full irrigation. Tables showing estimates for residual variance, genetic and marker variance, and other spatial hyper-parameters are given in the Online Resource 2.

We first focus on the results from fitting the models without genome-wide markers (models W1 and W1M), which are shown in Fig. 4a, b. The estimated genetic variances were similar within each trial except for the NoSpatial case which assigned all variation to the residual variance in the trial from 2011 with mild water stress (MWS), indicating a very bad model fit. Between the trials, there was more variation between the estimates of genetic variance; however, most 95$\%$ confidence intervals overlap between the different models and trials with a few exceptions. The uncertainty in the genetic variance was reduced when all trials were analysed together (W1M), which was expected as more data were used in this model. For the residual variance, we expected that it would differ both between models and trials as they described the amount of variation not explained by the structured model terms. As expected, the residual variance from NoSpatial was the largest as this model cannot explain spatial variation. The $\mathrm{AR1} \otimes \mathrm{AR1}$ model had the lowest residual variance, closely followed by the Matérn model in the 2011 trials. When all trials were analysed jointly, the residual variance increased slightly for the $\mathrm{AR1} \otimes \mathrm{AR1}$ and the Matérn.

We now focus on the results for models including genome-wide markers (models W2 and W2M) in Fig. 4c–e. We note that marker variance estimate had large uncertainty and was lower in 2011, particularly in the medium-water stress condition. The genetic variance not captured by markers (Fig. 4d) became more similar between the different trials compared to model W1 (as summarrized in Fig. 4a). The residual variance did not change significantly, indicating that the markers captured the variation that was already captured by the genetic effect modelled in W1 and W1M. However, with genome-wide markers we captured the genetic dependency between individuals with the model, which makes it possible to predict genetic value for non-phenotyped individuals as shown in the previous subsection.

**Fig. 5:**
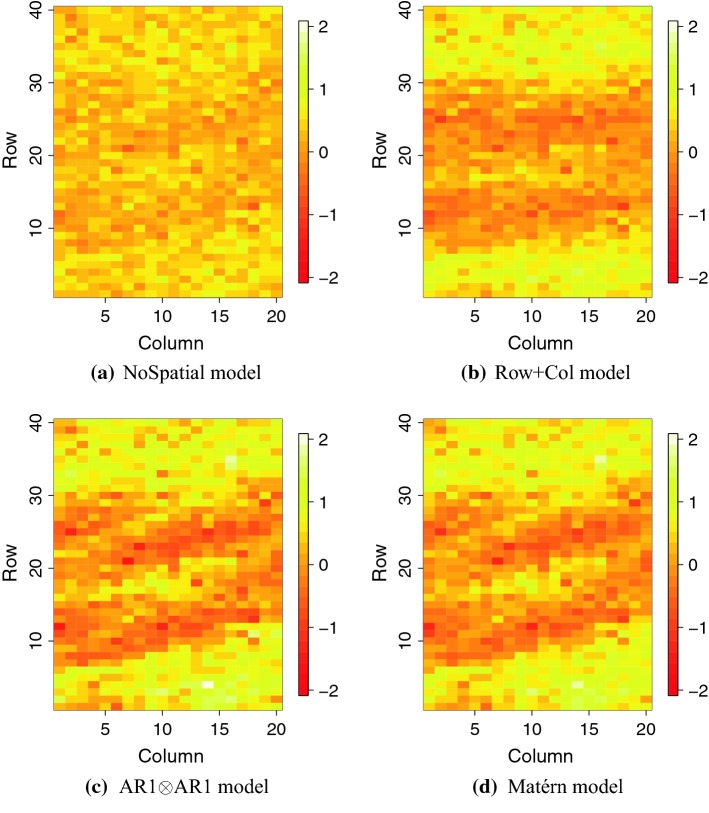
Posterior fitted values from the model W2 for trial 2011 FI using all three methods of spatial correction and no spatial correction

We show the fitted values from model W2 for the 2011 full irrigation trial in Fig. 5. These show how the $\mathrm{AR1} \otimes \mathrm{AR1}$ and Matérn models managed to capture the spatial pattern in the observations, whereas the NoSpatial model and $\mathrm{Row}+\mathrm{Col}$ model could not. Since we do not know the true spatial effects for the data, we cannot know for a fact that this spatial variability is real. However, from the simulation study we showed that the models accounting for spatial variability do not perform worse than the NoSpatial model when there is no spatial variability acting on the phenotype. Note that the scale here is different from the one in Fig. 1 since models were fitted to standardized data.

### Nelder wheel plot

**Fig. 6:**
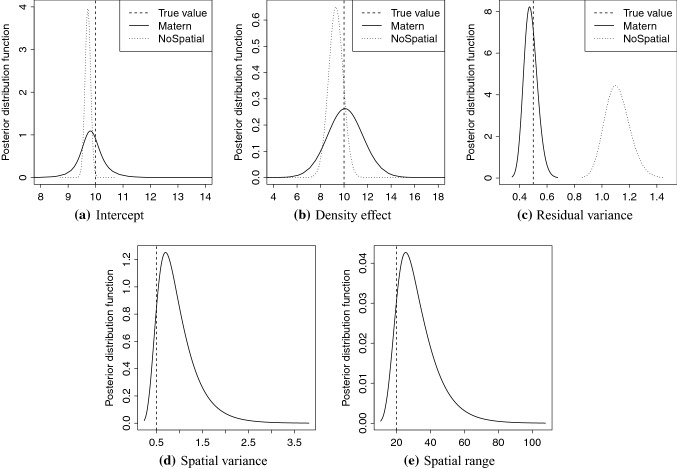
Posterior distributions from model fitted to simulated tree breeding data. Full and dotted curves represent the posterior distribution and the straight dashed line the true values

In this section, we present the results from fitting the model presented in the “Nelder wheel plot” section to the simulated tree breeding data. In Fig. 6, the posterior distributions for the intercept, fixed density effect, spatial range, spatial variance and residual variance from the Matérn model are presented along with the true values used in simulating the data. For all parameters, the posterior distribution contained the true values and the distribution modes were close to the true values for the Matérn model.

For the NoSpatial model, the true effect of density is barely covered by the 95$\%$ confidence interval of the posterior distribution (Fig. 6b), and the true intercept is not covered (Fig. 6a). The posterior residual variance is approximately twice as large as the true residual variance in Fig. 6c. This is expected as the NoSpatial model cannot account for the spatial variation, and we therefore expect it to perform worse than the Matérn model in this comparison.

**Fig. 7:**
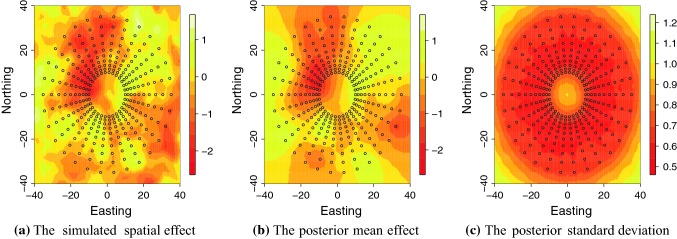
Simulated spatial effect, posterior mean spatial effect and posterior standard deviation of spatial effect in the Nelder wheel example. Black circles indicate tree positions

In Fig. 7, we show the simulated spatial effect, the posterior mean spatial effect and the standard deviation of the estimate. The mean estimate resembled closely the true spatial field, especially in locations where we had observations. The standard deviation was the smallest where we had observations and where the observations were more densely observed.

## Discussion

The objective of this paper was to test established spatial models for analysing agricultural field trials using the open-source R package INLA. We have fitted both spatial and genetic effects jointly in a simulated wheat trial data, a real wheat data set and a simulated tree breeding data set with the Nelder wheel design. Here, we highlight three points for discussion: (1) the importance of modelling spatial variation in agricultural field trials, (2) the flexibility of the R package INLA and the SPDE approach to model multiple trials and years as well as non-standard designs and non-standard phenotype distributions and (3) the limitations of the R package INLA to estimate large numbers of hyper-parameters and to fit genomic models.

### Modelling spatial variation

With the analysis of simulated wheat data sets, we showed that the estimates of genetic effects can be improved by accounting for spatial dependency in trials irrespective of the magnitude of the spatial variation. This is in line with the other studies (Elias et al. 2018; Rodríguez-Álvarez et al. 2018; Velazco et al. 2017; Piepho et al. 2008). We observed the greatest improvements with both the $\mathrm{AR1} \otimes \mathrm{AR1}$ model (Cullis and Gleeson 1991; Gilmour et al. 1997) and the Matérn model using the SPDE approach (Lindgren et al. 2011). We measured this improvement with the correlation and continuous rank probability score (CRPS) between the true and estimated effects as well as the average number of the top ten individuals that were among the 100 ranked individuals based on the estimates. When we attempted to model non-existing spatial variation, the results were not significantly worse compared to not modelling it. This observation suggests that the $\mathrm{AR1} \otimes \mathrm{AR1}$ model and the Matérn model are good default spatial models that do not overfit the data. A reviewer pointed out that field trial design and management effects should be modelled in addition to spatial effects. When this is required (e.g. Borges et al. 2019; González-Barrios et al. 2019), the demonstrated R package INLA can easily accommodate this via its general model formulae functionality, that is, by adding block and sub-block effects and row and column effects. These effects can be modelled either as fixed or as random effects.

### Flexibility of the R package INLA

Through modelling the real wheat data, we demonstrated the flexibility of the R package INLA to model both the genetic and spatial effects for several trials simultaneously. We treated the spatial variation in each trial as an independent realization of the chosen spatial model. By modelling spatial and genetic effects across several trials jointly with one model, we did not lose any information as we would if spatial effects were estimated first and then subtracted from the data (Schulz-Streeck et al. 2013). Furthermore, there is a large potential in modelling all trials jointly because this approach enables reduction of the required number of replicates per individual per trial and therefore test more individuals (Bernal-Vasquez et al. 2014). It also makes it possible to estimate location and year effects, which can be helpful for future management of the trial locations.

With the Nelder wheel design, we demonstrated the flexibility of the Matérn model using the SPDE approach with respect to the field trial design. This flexibility arises from the continuous modelling of spatial effects with the Matérn model as compared to the discrete approach of other standard models. The Nelder wheel example is a very special case and does not resemble standard agricultural field trials, which largely have a regular lattice layout of plots. We have nevertheless included this example to demonstrate the flexibility of the Matérn model and the R package INLA. This approach can be used for regular as well as non-regular designs, which can be useful in special settings, for example, when plot sizes differ (Archbold et al. 1987), when design is non-standard as in the Nelder wheel design (Parrott et al. 2012), when spatial correlation is not expected to follow standard patterns due to external variation (Bakka et al. 2019), or if the terrain does not allow for a lattice-like layout of plots. Another possible use of the Matérn model could be to jointly model neighbouring trials. In this case, the Matérn model can accommodate any layout of the plots across the trials, while the $\mathrm{AR1} \otimes \mathrm{AR1}$ model would require that plots from the neighbouring trials follow a common layout to all trials. Other applications of the Matérn model and the SPDE approach could be in conservation and utilization of genetic resources in forestry, particularly in natural or semi-natural stands not planted in a formal layout, and for identification of trees in the wild for collection of seed for cultivation or for reforestation. The approach can also make use of area observations (Lindgren et al. 2011; Bakka et al. 2018) to model total yield per area with varying area between plots. These flexibilities could enable design of new field trials or an advanced analysis of existing trials that do not follow the common lattice-like layout.

In this study, we focused on phenotypes that can be modelled with a Gaussian distribution only. However, the R package INLA enables seamless modelling of other distributions such as binomial, Poisson and others. Breeder’s scores and other types of field trial data frequently follow these types of distributions. For most models, the only code change required is a switch of the distribution family; for example, to change the model with a continuous Gaussian distribution to a discrete Poisson distribution we simply change inla(..., family = ”Gaussian”) to inla(..., family = ”Poisson”). Krainski et al. (2018) or Blangiardo and Cameletti (2015) provide further details on this. While the code change is simple, we have to note that the change of phenotype model impacts the interpretation of parameters. To this end, the R package INLA enables sampling from posterior distributions and these samples can be used to calculate parameters of interest. De Villemereuil et al. (2016) provide an excellent overview of this topic.

### Limitations of the R package INLA

While the R package INLA enables flexible modelling of data from multiple trials and years, this might usually require increasing the model complexity by accounting for trial-specific residual variance or trial-specific spatial parameters—by increasing the number of hyper-parameters, that is, parameters controlling the likelihood and latent field, for example variance parameters. We have performed such an analysis with the real wheat data, where spatial variation in 2011 and 2012 trials differed substantially in both dependency with distance and variance. While this can be accommodated with the R package INLA, we highlight that the INLA method is best when it is based on a relative small number of non-Gaussian hyper-parameters, typically less than ten, and not exceeding 20. This limitation is due to the numerical integration of multidimensional posterior distribution of hyper-parameters in INLA (Rue et al. 2017). Since there is limited information to estimate hyper-parameters from a single trial, a parsimonious solution would be to group similar trials together and estimate hyper-parameters per group instead of per trial. This is what we did for the 2011 and 2012 trials with the real wheat data.

The main drawback with using R package INLA for analysing modern agricultural trials is that genome-wide marker data are highly dimensional, which leads to dense systems of equations. INLA is based on numerical approximations and numerical methods for sparse matrices, and even though INLA can fit genomic models either via the genomic relationship matrix or via marker effects (Strandén and Garrick 2009), there is substantial computational overhead to handle such models, which is not the case for the pedigree model which has a sparse precision matrix (Steinsland and Jensen 2010; Henderson et al. 1984). This is why we chose to fit the genome-wide markers directly via the principal component approach, which is similar to the proposal of Ødegård et al. (2018). Another option would be to fit a model with individual genetic effects following VanRaden (2008), but with a genomic relationship matrix that uses dense–sparse partitioning into core and non-core individuals (Misztal 2016). More research is required in this area to increase the usefulness of the R package INLA for the modern breeding applications.

Finally, since the INLA method implements a full Bayesian analysis, prior distributions have to be set for all parameters of the model. The marker variance estimates in the models for Chilean wheat data were quite small, and we expected this to be larger. Testing the same models using the informative penalized complexity priors (Simpson et al. 2017) increased the mean marker variance. However, we have used the default prior distributions in the R package INLA for simplicity. It should be emphasized that using default priors is a choice as much as using any other prior or even using a specific distribution for the phenotype observations. Setting a prior based on the knowledge about the process is likely to improve the inference. Choosing a prior distribution for parameters in the model is not always straightforward, and more work is being done in the statistics community to improve this (Fuglstad et al. 2019).

## Conclusion

This study showed how to fit established spatial models for analysing agricultural field trials using the open-source R package INLA. The results from the simulation study showed higher accuracy when spatial dependency was modelled and the highest increase in accuracy was reached using the discrete autoregressive ($\mathrm{AR1} \otimes \mathrm{AR1}$) model and the continuous Gaussian random field (Matérn) model. Both models can be seamlessly fitted with the R package INLA, including joint modelling of multiple trials. The Matérn model and SPDE approach provide a flexibility with respect to field design that is not obviously available elsewhere and are particularly suitable for agricultural field trials that do not have a standard lattice-like structure such as the Nelder wheel design used in tree breeding. This flexibility opens opportunities for new field trial designs. It is freely available and yields interpretable parameters for the estimated spatial effects.

## Electronic supplementary material

Below is the link to the electronic supplementary material.

Supplementary material 1 (zip 4034 KB)

Supplementary material 2 (pdf 182 KB)
