# Supplementary material for: Flexible modelling of spatial variation in agricultural field trials with the R package INLA
Source: Theor Appl Genet. 2019 Sep 18;132(12):3277–93. doi: 10.1007/s00122-019-03424-y (PMC6820601; doi:10.1007/s00122-019-03424-y)
Supplement: Supplementary file 2 — Supplementary material 2 (pdf 182 KB) [file 122_2019_3424_MOESM2_ESM.pdf]

Theoretical and Applied Genetics manuscript No.
(will be inserted by the editor)

Modelling spatial variation in agricultural field trials with INLA - Online Resource 2

Maria Lie Selle · Ingelin Steinsland · John M. Hickey · Gregor Gorjanc

Received: date / Accepted: date

1 Simulation study

1.1 Accuracy for models fitted to data from AR1⊗AR1

This section presents the results from the simulation study based on data where the spatial effects are from the AR1⊗AR1 model. Here, we present the average correlation and CRPS between the true and estimated breeding value, and using the number of the top ten individual that were among the top 100 ranked individuals when considering estimated breeding value (posterior mean). Table 1, Table 2, and Table 3 present the correlation, CRPS and ranking respectively from the preliminary yield trial. Table 4, Table 5, and Table 6 present the correlation, CRPS and ranking respectively for the 1000 doubled-haploid individuals that were genotyped, but not phenotyped.

1.2 Posterior hyperparameters from data with

In this section, we present the average posterior mean with standard error for hyper-parameters in the simulation study. The parameters we present are the residual variance σ_e^2, genetic σ_g^2 and marker variance $\sigma_{u^*}^2$, and additional

The Research Council of Norway, Grant number: 250362; The UK Biotechnology and Biological Sciences Research Council, Grant numbers: BB/L020467/1 and BBS/E/D/30002275

M.L. Selle · I. Steinsland
Department of Mathematical Sciences, Norwegian University of Science and Technology (NTNU), Trondheim, Norway
E-mail: maria.selle@ntnu.no

J. M. Hickey · G. Gorjanc
The Roslin Institute and Royal (Dick) School of Veterinary Studies, University of Edinburgh, Easter Bush, UK

Table 1 Correlation between the simulated true and estimated breeding value in the preliminary yield trial by the proportion of spatial variation, the spatial model and using genome-wide markers. Spatial variation from an AR1⊗AR1 model. The standard error was around 0.002

Genome-wide markers	No			Yes		
Prop. of spatial var	0.00	0.50	0.75	0.00	0.50	0.75
NoSpatial	0.39	0.38	0.38	0.61	0.61	0.61
Row+Col	0.39	0.39	0.40	0.61	0.62	0.62
AR1⊗AR1	0.39	0.43	0.49	0.61	0.65	0.69
Matérn	0.39	0.43	0.48	0.61	0.65	0.69

Table 2 CRPS between the simulated true and estimated breeding value in the preliminary yield trial by the proportion of spatial variation, the spatial model and using genome-wide markers. Spatial variation from an AR1⊗AR1 model. The standard error was around 0.0002

Genome-wide markers	No			Yes		
Prop. of spatial var	0.00	0.50	0.75	0.00	0.50	0.75
NoSpatial	0.149	0.148	0.147	0.114	0.112	0.112
Row+Col	0.169	0.144	0.141	0.114	0.111	0.110
AR1⊗AR1	0.169	0.131	0.122	0.114	0.108	0.102
Matérn	0.148	0.132	0.123	0.114	0.108	0.103

Table 3 Average number of the top ten individuals among the top 100 ranked individuals in the preliminary yield trial by the proportion of spatial variation, the spatial model and using genome-wide markers. Spatial variation from an AR1⊗AR1 model. The standard error was around 0.05

Genome-wide markers	No			Yes		
Prop. of spatial var	0.00	0.50	0.75	0.00	0.50	0.75
NoSpatial	3.89	3.82	3.88	6.32	6.29	6.46
Row+Col	3.89	3.90	4.06	6.32	6.39	6.65
AR1⊗AR1	3.89	4.38	5.02	6.32	6.88	7.57
Matérn	3.89	4.35	4.92	6.32	6.83	7.47

Table 4 Correlation between the true and predicted breeding value for the non-phenotyped doubled-haploid lines by the proportion of spatial variation and the spatial model. Spatial variation from an AR1⊗AR1 model. The standard error was around 0.004

Prop. of spatial var	0.00	0.50	0.75
NoSpatial	0.36	0.36	0.36
Row+Col	0.36	0.36	0.37
AR1⊗AR1	0.36	0.39	0.43
Matérn	0.36	0.38	0.42

Table 5 CRPS between the true and predicted breeding value for the non-phenotyped doubled-haploid lines by the proportion of spatial variation and the spatial model. Spatial variation from an AR1⊗AR1 model. The standard error was around 0.00004

Prop. of spatial var	0.00	0.50	0.75
NoSpatial	0.128	0.125	0.125
Row+Col	0.128	0.125	0.124
AR1⊗AR1	0.128	0.124	0.121
Matérn	0.128	0.124	0.121

Table 6 Average number of the top ten individuals among the top 100 ranked individuals for the non-phenotyped doubled-haploid lines by the proportion of spatial variation and the spatial model. Spatial variation from an AR1⊗AR1 model. The standard error was around 0.06

Prop. of spatial var	0.00	0.50	0.75
NoSpatial	0.97	3.31	3.33
Row+Col	0.98	3.36	3.46
AR1⊗AR1	0.97	3.62	4.11
Matérn	0.97	3.60	4.02

parameters in each of the spatial models presented in the paper. For the NoSpatial model parameters are given in Table 7, for the Row+Col model see Table 8, for the AR1⊗AR1 see Table 9, and for the SPDE approach see Table 10.

We remove the results from inference on a model if numerical instabilities occur. By numerical instabilities we mean posterior hyperparameters that are obviously too big. This was the case with a few models with data sets having no spatial variation.

Table 7 Average posterior mean and standard error of parameters in the simulation study using the NoSpatial model

	σ_e^2	SE(σ_e^2)	σ_g^2	SE(σ_g^2)	σ_{u*}^2	SE(σ_{u*}^2)
0.00	0.730	0.006	0.041	0.001		
0.50	0.717	0.006	0.041	0.001		
0.75	0.729	0.006	0.042	0.001		
0.00	0.712	0.006			9e-06	7e-08
0.50	0.701	0.006			9e-06	8e-08
0.75	0.712	0.006			9e-06	8e-08

σ_e^2 - residual variance; σ_g^2 - genetic variance (σ_n^2 - non-additive genetic variance, when the model includes genome-wide marker effects); σ_{u*}^2 - marker variance;

Table 8 Average posterior mean and standard error of parameters in the simulation study using the Row+Col model

	σ_e^2	SE(σ_e^2)	σ_g^2	SE(σ_g^2)	$\sigma_{u^*}^2$	SE($\sigma_{u^*}^2$)	σ_r^2	SE(σ_r^2)	σ_c^2	SE(σ_c^2)
0.00	0.745	0.006	0.025	0.001			2e-04	3e-05	3e-04	3e-05
0.50	0.618	0.006	0.046	0.001			0.046	0.001	0.051	0.001
0.75	0.572	0.005	0.049	0.001			0.072	0.001	0.081	0.001
0.00	0.712	0.006			9e-06	7e-08	3e-05	3e-05	3e-05	3e-05
0.50	0.605	0.005			9e-06	8e-08	0.046	0.001	0.051	0.001
0.75	0.563	0.005			9e-06	8e-08	0.072	0.001	0.081	0.001

σ_e^2 - residual variance; σ_g^2 - genetic variance (σ_n^2 - non-additive genetic variance, when the model includes genome-wide marker effects); $\sigma_{u^*}^2$ - marker variance; σ_r^2 - row variance; σ_r^2 - column variance

Table 9 Average posterior mean and standard error of parameters in the simulation study using the AR1xAR1 model

	σ_e^2	SE(σ_e^2)	σ_g^2	SE(σ_g^2)	$\sigma_{u^*}^2$	SE($\sigma_{u^*}^2$)	σ_r^2	SE(σ_r^2)	ρ_r	SE(ρ_r)	ρ_c	SE(ρ_r)
0.00	0.744	0.006	0.026	0.001			0.001	2e-04	0.013	0.003	0.011	0.003
0.50	0.344	0.003	0.054	0.001			0.312	0.003	0.651	0.001	0.821	0.001
0.75	0.171	0.002	0.062	0.001			0.425	0.004	0.606	0.001	0.823	0.001
0.00	0.712	0.006			9e-06	7e-08	0.001	2e-04	0.026	0.004	0.028	0.004
0.50	0.343	0.003			8e-06	8e-08	0.310	0.003	0.650	0.001	0.821	0.001
0.75	0.177	0.002			8e-06	8e-08	0.422	0.004	0.601	0.001	0.824	0.001

σ_e^2 - residual variance; σ_g^2 - genetic variance (σ_n^2 - non-additive genetic variance, when the model includes genome-wide marker effects); $\sigma_{u^*}^2$ - marker variance; σ_r^2 - plot variance; ρ_r - row auto-correlation; ρ_c - column auto-correlation

Table 10 Average posterior mean and standard error of parameters in the simulation study using the SPDE approach

	σ_e^2	SE(σ_e^2)	σ_g^2	SE(σ_g^2)	$\sigma_{u^*}^2$	SE($\sigma_{u^*}^2$)	σ_s^2	SE(σ_s^2)	ρ	SE(ρ)
0.00	0.726	0.006	0.042	0.001			0.011	0.002	7.956	0.422
0.50	0.356	0.003	0.053	0.001			0.357	0.003	10.204	0.045
0.75	0.180	0.002	0.062	0.001			0.537	0.005	10.162	0.038
0.00	0.711	0.006			9e-06	7e-08	0.008	0.002	8.094	0.448
0.50	0.354	0.003			8e-06	8e-08	0.357	0.003	10.230	0.045
0.75	0.186	0.002			8e-06	8e-08	0.537	0.005	10.153	0.037

σ_e^2 - residual variance; σ_g^2 - genetic variance (σ_n^2 - non-additive genetic variance, when the model includes genome-wide marker effects); $\sigma_{u^*}^2$ - marker variance; σ_s^2 - spatial variance; ρ - spatial range

2 Chilean wheat data

In this section we present the posterior mean and corresponding 95% credible interval for the hyper-parameters in models W1, W2, W1M and W2M for the trials in real wheat data. These are given in Table 11, Table 12, Table 13 and Table 14 respectively for the NoSpatial, Row+Col, AR1⊗AR1 models and the

SPDE approach. Note that in models W1M and W2M there are two sets of spatial hyper-parameters, the first row for the 2011 trials and the second for 2012 trials.

Table 11 Posterior mean and 95% confidence interval of hyper-parameters from the NoSpatial spatial model by trial and genetic model

Trial, model	σ_e^2	95%CI	σ_g^2	95%CI	σ_{u*}^2	95%CI
11-FI,W2	0.271	(0.236,0.312)	0.122	(0.085,0.166)	2e-04	(0,0.001)
11-FI,W1	0.271	(0.236,0.312)	0.122	(0.084,0.165)		
11-MWS,W2	0.613	(0.556,0.676)	1e-04	(0,7e-04)	1e-04	(0,7e-04)
11-MWS,W1	0.613	(0.556,0.676)	1e-04	(0,7e-04)		
12-FI,W2	0.102	(0.089,0.118)	0.066	(0.048,0.089)	0.006	(8e-04,0.015)
12-FI,W1	0.102	(0.089,0.117)	0.076	(0.059,0.097)		
12-MWS,W2	0.086	(0.074,0.098)	0.061	(0.043,0.082)	0.014	(0.005,0.027)
12-MWS,W1	0.085	(0.074,0.098)	0.084	(0.067,0.104)		
W2M	0.278	(0.264,0.293)	0.058	(0.047,0.074)	0.002	(2e-04,0.005)
W1M	0.278	(0.264,0.293)	0.061	(0.049,0.076)		

σ_e^2 - residual variance; σ_g^2 - genetic variance (σ_n^2 - non-additive genetic variance, when the model includes genome-wide marker effects); σ_{u*}^2 - marker variance

Table 12 Posterior mean and 95% confidence interval of hyper-parameters from the Row+Col spatial model by trial and genetic model

Trial, model	σ_e^2	95%CI	σ_g^2	95%CI	σ_{u*}^2	95%CI	σ_r^2	95%CI	σ_c^2	95%CI
11-FI,W2	0.134	(0.115,0.155)	0.085	(0.060,0.116)	0.006	(7e-04,0.017)	0.155	(0.096,0.240)	0.003	(1e-04,0.010)
11-FI,W1	0.134	(0.116,0.156)	0.097	(0.073,0.124)			0.153	(0.095,0.240)	0.002	(1e-04,0.008)
11-MWS,W2	0.155	(0.134,0.179)	0.075	(0.053,0.101)	2e-04	(0,8e-04)	0.390	(0.247,0.599)	1e-04	(0,7e-04)
11-MWS,W1	0.155	(0.134,0.179)	0.075	(0.053,0.101)			0.391	(0.247,0.598)	1e-04	(0,7e-04)
12-FI,W2	0.057	(0.049,0.066)	0.080	(0.063,0.101)	0.007	(0.002,0.016)	0.039	(0.023,0.061)	0.002	(-0.001,0.027)
12-FI,W1	0.056	(0.049,0.066)	0.093	(0.076,0.111)			0.038	(0.023,0.061)	0.004	(-0.001,0.057)
12-MWS,W2	0.046	(0.040,0.053)	0.061	(0.047,0.078)	0.011	(0.005,0.022)	0.0404	(0.025,0.064)	9e-04	(-1e-04,0.009)
12-MWS,W1	0.046	(0.040,0.053)	0.082	(0.067,0.098)			0.041	(0.025,0.064)	7e-04	(0,0.006)
W2M	0.129	(0.122,0.136)	0.052	(0.042,0.064)	0.002	(1e-04,0.006)	0.275<br>0.034	(0.1978,0.3735)<br>(0.023,0.050)	0.002<br>1e-04	(3e-04,0.005)<br>(1e-05,5e-04)
W1M	0.129	(0.122,0.136)	0.056	(0.046,0.067)			0.275<br>0.034	(0.198,0.374)<br>(0.023,0.049)	0.002<br>1e-04	(3e-04,0.005)<br>(1e-05,5e-04)

σ_e^2 - residual variance; σ_g^2 - genetic variance (σ_n^2 - non-additive genetic variance, when the model includes genome-wide marker effects); σ_{u*}^2 - marker variance; σ_r^2 - row variance; σ_r^2 - column variance

Table 13 Posterior mean and 95% confidence interval of hyper-parameters from the AR1⊗AR1 spatial model by trial and genetic model

Trial, model	σ_e^2	95%CI	σ_g^2	95%CI	$\sigma_{u^*}^2$	95%CI	σ_r^2	95%CI	ρ_r	95%CI	ρ_c	95%CI
11-FI,W2	0.032	(0.023,0.044)	0.093	(0.075,0.117)	4e-04	(0,0.002)	0.171	(0.131,0.221)	0.604	(0.499,0.695)	0.863	(0.813,0.904)
11-FI,W1	0.032	(0.023,0.043)	0.098	(0.081,0.118)			0.172	(0.132,0.222)	0.604	(0.499,0.695)	0.864	(0.814,0.904)
11-MWS,W2	0.023	(0.016,0.031)	0.071	(0.058,0.086)	2e-04	(0,8e-04)	0.235	(0.178,0.307)	0.663	(0.564,0.745)	0.907	(0.872,0.937)
11-MWS,W1	0.023	(0.016,0.031)	0.0711	(0.058,0.086)			0.235	(0.178,0.308)	0.662	(0.563,0.744)	0.907	(0.872,0.937)
12-FI,W2	0.048	(0.040,0.057)	0.082	(0.065,0.102)	0.006	(0.001,0.015)	0.048	(0.030,0.072)	0.266	(2e-04,0.518)	0.966	(0.933,0.987)
12-FI,W1	0.047	(0.039,0.057)	0.093	(0.077,0.112)			0.048	(0.031,0.072)	0.273	(0.011,0.523)	0.965	(0.930,0.986)
12-MWS,W2	0.036	(0.030,0.043)	0.063	(0.049,0.080)	0.011	(0.005,0.022)	0.056	(0.035,0.088)	0.388	(0.104,0.636)	0.970	(0.943,0.988)
12-MWS,W1	0.035	(0.029,0.043)	0.083	(0.069,0.099)			0.056	(0.035,0.087)	0.369	(0.090,0.617)	0.969	(0.941,0.987)
W2M	0.067	(0.062,0.072)	0.050	(0.041,0.061)	0.003	(5e-04,0.007)	0.210	(0.172,0.256)	0.667	(0.601,0.729)	0.897	(0.870,0.921)
							0.050	(0.036,0.067)	0.237	(0.026,0.439)	0.968	(0.945,0.983)
W1M	0.067	(0.062,0.071)	0.056	(0.047,0.066)			0.210	(0.172,0.257)	0.668	(0.601,0.729)	0.898	(0.870,0.922)
							0.050	(0.036,0.067)	0.236	(0.021,0.433)	0.967	(0.944,0.983)

σ_e^2 - residual variance; σ_g^2 - genetic variance (σ_n^2 - non-additive genetic variance, when the model includes genome-wide marker effects); $\sigma_{u^*}^2$ - marker variance; σ_r^2 - plot variance; ρ_r - row auto-correlation; ρ_c - column auto-correlation

Table 14 Posterior mean and 95% confidence interval of hyper-parameters from the SPDE spatial model by trial and genetic model

Trial, model	σ_e^2	95%CI	σ_g^2	95%CI	$\sigma_{u^*}^2$	95%CI	σ_s^2	95%CI	ρ	95%CI
11-FI,W2	0.061	(0.048,0.076)	0.085	(0.066,0.109)	0.003	(1e-04,0.009)	0.237	(0.142,0.375)	11.828	(8.245,16.685)
11-FI,W1	0.060	(0.047,0.076)	0.092	(0.073,0.114)			0.238	(0.143,0.377)	11.845	(8.273,16.754)
11-MWS,W2	0.0345	(0.025,0.047)	0.069	(0.056,0.086)	2e-04	(0,8e-04)	0.467	(0.267,0.772)	14.493	(10.157,20.279)
11-MWS,W1	0.0346	(0.025,0.047)	0.070	(0.056,0.086)			0.474	(0.266,0.791)	14.623	(10.165,20.556)
12-FI,W2	0.0809	(0.0681,0.096)	0.073	(0.055,0.096)	0.005	(8e-04,0.015)	0.020	(0.009,0.0409)	14.271	(5.878,29.896)
12-FI,W1	0.080	(0.067,0.095)	0.083	(0.066,0.103)			0.0206	(0.009,0.0406)	13.530	(5.615,28.692)
12-MWS,W2	0.063	(0.053,0.073)	0.064	(0.048,0.085)	0.013	(0.005,0.026)	0.036	(0.014,0.081)	19.638	(8.964,40.524)
12-MWS,W1	0.062	(0.053,0.073)	0.087	(0.071,0.106)			0.033	(0.014,0.068)	17.123	(7.886,34.763)
W2M	0.097	(0.091,0.103)	0.048	(0.039,0.060)	0.003	(4e-04,0.007)	0.413<br>0.023	(0.262,0.642)<br>(0.011,0.044)	16.989<br>17.635	(12.846,22.693)<br>(9.054,31.149)
W1M	0.097	(0.091,0.103)	0.054	(0.045,0.064)			0.415<br>0.0216	(0.263,0.646)<br>(0.011,0.039)	17.079<br>16.247	(12.906,23.044)<br>(8.808,28.610)

σ_e^2 - residual variance; σ_g^2 - genetic variance (σ_n^2 - non-additive genetic variance, when the model includes genome-wide marker effects); $\sigma_{u^*}^2$ - marker variance; σ_s^2 - spatial variance; ρ - spatial range
